# Artificial intelligence-enhanced infrared thermography as a diagnostic tool for thyroid malignancy detection

**DOI:** 10.1080/07853890.2024.2425826

**Published:** 2024-11-08

**Authors:** Panpicha Chantasartrassamee, Boonsong Ongphiphadhanakul, Ronnarat Suvikapakornkul, Panus Binsirawanich, Chutintorn Sriphrapradang

**Affiliations:** a Division of Endocrinology and Metabolism, Department of Medicine, Faculty of Medicine Ramathibodi Hospital, Mahidol University, Bangkok, Thailand; b Breast and Endocrine Surgery Unit, Department of Surgery, Faculty of Medicine Ramathibodi Hospital, Mahidol University, Bangkok, Thailand; c Department of Otolaryngology, Faculty of Medicine Ramathibodi Hospital, Mahidol University, Bangkok, Thailand

**Keywords:** Convolutional neural networks, diagnostic imaging, machine learning, neoplasm, thermography, thyroid nodule

## Abstract

**Introduction:**

Thyroid nodules are common, and investigation is crucial for excluding malignancy. Increased intranodular vascularity is frequently observed in malignant tumors, which can be detected through increased skin surface temperatures using noninvasive infrared thermography. We aimed to develop a diagnostic tool for thyroid cancer using infrared thermal images combined with an artificial intelligence (AI) algorithm.

**Methods:**

We conducted a prospective cross-sectional study involving participants with thyroid nodules undergoing thyroid surgery. Infrared thermal images were collected using a thermal camera on the day prior to surgery. In combination with the final thyroid pathological reports, we utilized a machine learning model based on the pre-trained ResNet50V2 model, a convolutional neural network, to evaluate diagnostic accuracy for malignancy diagnosis.

**Results:**

The study included 98 participants, 58 with malignant thyroid nodules and 40 with benign thyroid nodules, as determined by pathological results. The AI-enhanced infrared thermal image analyses demonstrated good performance in distinguishing between benign and malignant thyroid nodules, achieving an accuracy of 75% and a sensitivity of 78%. These parameters were slightly lower than those of the AI-model predictor that integrated current practice using preoperative thyroid ultrasound findings and cytological results, yielding an accuracy of 81% and a sensitivity of 84%.

**Conclusions:**

The infrared thermal images, assisted by an AI model, exhibit good performance in distinguishing thyroid malignancy from benign nodules. This imaging modality has great potential to be used as a noninvasive screening tool for adjunct evaluation of thyroid nodules.

## Introduction

Thyroid nodules are prevalent in the adult population, with detection rates varying: 2%–6% (palpation), 19%–35% (ultrasound) and 8%–65% (autopsy) [1–3]. While most thyroid nodules are benign, the primary purpose of evaluating thyroid nodules is to exclude malignancy, which occurs in 7%–15% of all thyroid nodules [4–7]. This prevalence of thyroid malignancy is higher than the historical estimate of 5% [8], likely due to increased detection of asymptomatic thyroid nodules found incidentally during cross-sectional imaging performed for other reasons. The assessment of malignancy risk involves clinical risk factors and sonographic findings [9, 10]. In addition, most patients require fine-needle aspiration biopsy (FNAB) to evaluate the risk of thyroid malignancy [9, 10]. This risk stratification improves the detection of thyroid malignancy with high sensitivity and specificity, while reducing unnecessary FNAB procedures or surgeries [11]. Although the FNAB is a relatively simple procedure, it remains invasive, and its nondiagnostic rates depend on the performer’s skill and experience [12]. Thyroid ultrasound is a noninvasive mandatory step in nodule evaluation but is operator-dependent. Several techniques have been proposed as adjunctive tools to improve the detection of thyroid malignancy, including infrared thermal imaging [13].

Thermal imaging is the process of converting infrared radiation (heat) into visible images, revealing temperature differences in a scene captured by a thermal camera. Typically, malignant cells require nutrients, leading to increased angiogenesis [14]. This vascular proliferation results in higher local temperatures, allowing the differentiation of thyroid cancers from benign nodules. Due to its superficial position on the neck, the thyroid gland’s temperature is easily measurable. Infrared thermal imaging may assist in the detection of these malignant thyroid tumors [13, 15, 16]. This imaging technique has been previously applied to skin cancer and breast cancer detection [17, 18].

Artificial intelligence (AI) refers to the field of computer science dedicated to developing algorithms and datasets that enable problem-solving capabilities. Within AI, machine learning is a key process that utilizes algorithms and statistical models to achieve various tasks [19, 20]. Deep learning, a subtype of machine learning, focuses on training artificial neural networks to perform tasks without explicit programming. Machine learning algorithms have been widely applied for medical-related problems. However, there has been limited research on utilizing machine learning methods for the detection of thyroid malignancy using infrared thermal images. The study aims to develop an AI model by using thyroid infrared thermal images to aid in adjunct thyroid malignancy diagnosis.

## Methods

### Study design and population

This was a prospective cross-sectional study on patients with thyroid nodules who consecutively underwent for thyroid surgery at the otolaryngology and surgery inpatient ward of Ramathibodi Hospital during January and October 2023. We included patients aged 18 years or older who were diagnosed with thyroid nodules and had FNAB cytologic results prior to surgery. The decision to perform a thyroidectomy was primarily made by the surgeons, considering a combination of clinical risk factors, FNAB results, imaging studies, and patient-specific factors, including patient preference. Patients with a body temperature exceeding 37.5 °C, active infection or inflammation in the cervical region, or a history of previous thyroid surgery were excluded. All patients provided written informed consent to participate in the study. Written informed consent to publish the identifying details of individual participants has been obtained from all individuals (or their legal guardian). All efforts to anonymous individuals should still be taken. The study protocol was approved by the Human Research Ethics Committee of the Faculty of Medicine Ramathibodi Hospital, Mahidol University (MURA2022/601).

### Data collection

All participants underwent thyroid infrared thermography on the day of admission before undergoing surgery. Subsequently, they underwent surgery following the standard treatment care.

Patient data, including critical variables such as age, sex, weight, height and body temperature, thyroid-stimulating hormone (TSH) levels, neck ultrasonographic images of thyroid nodules, cytological results of thyroid nodules, and postoperative pathological results of thyroid nodules, were obtained from electronic medical record. Pathology is the gold standard for differentiating between benign and malignant thyroid nodules.

### Thyroid infrared thermographic imaging protocol

**Figure 1.**
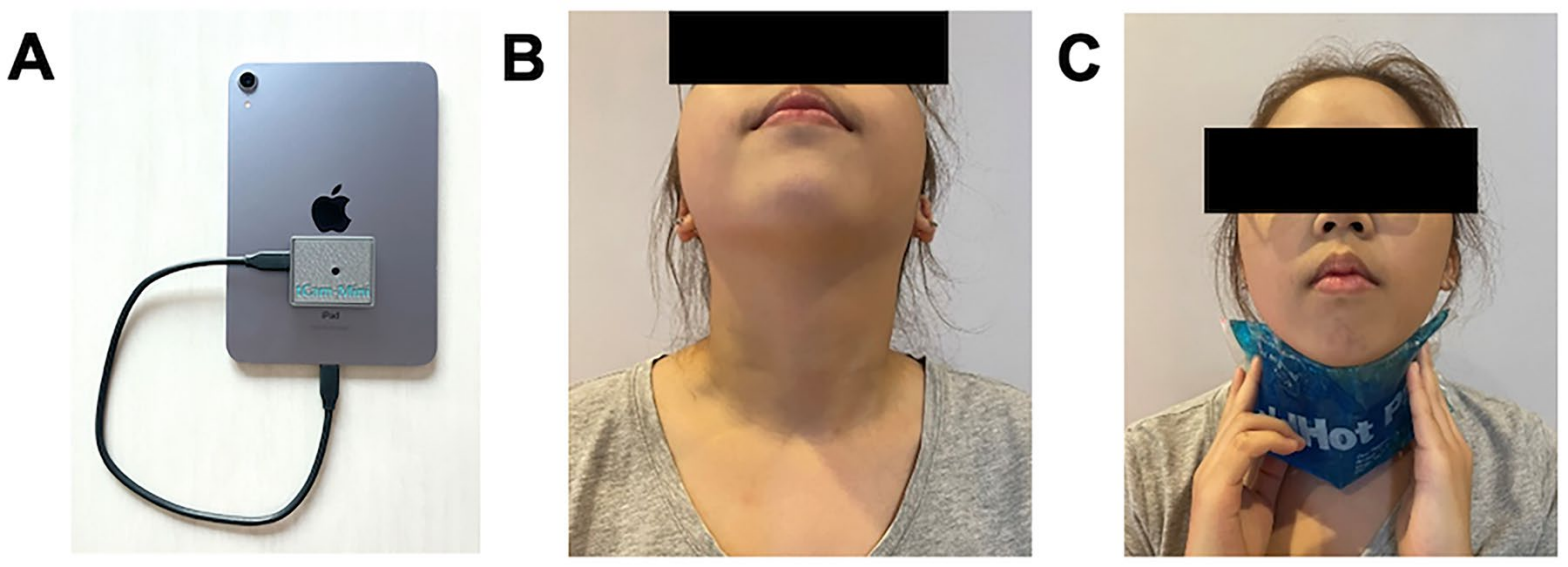
(A) tCam-mini thermal camera connected to iPad mini (6th generation), (B) patient positioning during thyroid infrared thermal imaging, and (C) application of cooling gel on the anterior neck region.

Thyroid infrared thermographic images were captured using the tCam-mini thermal camera (danjuliodesigns LLC, Boulder, CO), which was connected to a 6th generation iPad mini (Apple Inc, Cupertino, CA) (Figure 1A). The tCam-mini is a thermal and color camera designed for use with smartphones. The images captured by the tCam-mini were exported through the ‘tCamView’ application (danjuliodesigns LLC, Boulder, CO), with settings configured to 320 × 240 pixels and an operating temperature range of 25–40 °C. The images were captured using a ‘white-hot’ palette, where brighter areas corresponded to higher temperatures, as visually represented in black and white images.

The protocol for this study was adapted from the dynamic infrared thermography protocol in a previous study [15]. To enhance measurement reliability and control for external factors, all thermographic images were captured by a single trained operator to minimize variability. Patients were instructed to refrain from eating, drinking, or applying any creams, oils, or chemical substances to the neck region for at least 30 min prior to imaging. Room temperature was maintained between 21 and 25 °C for at least 10 min after the patient stayed in the room. Patients were initially positioned seated to minimize potential displacement and were instructed to tilt their heads slightly back and looking up during imaging to ensure consistent angles and exposure (Figure 1B). The distance between the patients and the camera was approximately 60 cm. An initial image was captured before applying cooling. A cooling gel (estimate to be at 8 °C) was then applied to the neck region for 30 s (Figure 1C), followed by the capture of subsequent images every 15 s over a 5-min period after removing the cooling gel, resulting in a sequence of 22 images for each participant.

### Convolutional neural network architecture

The pre-trained ResNet50V2 model, one of the convolutional neural networks, was used in deep learning algorithm for infrared thermal image recognition to differentiate between benign and malignant thyroid nodules. The ResNet50V2 model comprises 50 layers, including convolutional, batch normalization, activation, and pooling layers. This model is a saved network that was previously trained from ImageNet database. We used transfer learning to fine-tune the ResNet50V2 model by replacing its final fully connected layers with new layers consisting of dropout, batch normalization, and dense layers. These new layers were trained on our dataset, while the weights of the pre-trained layers were frozen to preserve the learned features.

### Data set and cross validation

The data set consists of 22 thermal images from each participant, with the images allocated into an 80% training set and a 20% validation set. We applied five-fold cross validation techniques to evaluate the model on the data set.

### Statistical analyses

Descriptive statistics were reported as mean and standard deviations (SD) for normally distributed data, while median and interquartile ranges (IQR) were used for non-parametric data. Categorical data were presented as absolute numbers (n) and relative frequencies (%). The Chi-square test was applied to evaluate the association of qualitative variables. Statistical significance was considered for p-values less than 0.05. Data analysis was performed using STATA Software version 15.0 (StataCorp LLC, College Station, TX). Given the exploratory nature of this pilot study, a formal sample size calculation was not conducted.

The evaluation metrics used to analyze the performance of the machine learning model included accuracy, recall, precision, F1 score, area under Receiver Operating Characteristic (ROC) curve, the Matthews correlation coefficient (MCC), and Kappa hat coefficient (Kappa). The calculated parameters of these metrics are based on true positive (TP), true negative (TN), false positive (FP), and false negative (FN) rates, as shown in Table 1. Accuracy is a metric that measure how effectively a model predicts actual values. Higher accuracy values typically indicate better performance. Recall (or true positive rate, sensitivity) represents the percentage of correctly identified positive instances out of the total real positive instances. Precision (or positive predictive value) indicates the accuracy of the model’s positive predictions, with higher values suggesting more accurate positive predictions. The F1 score serves as a valuable metric that balances between precision and recall, with a range from 0 to 1; a higher value indicates better model performance. MCC is a statistical measure that assesses the overall performance of a model, ranging from −1 to 1. A higher MCC value suggests a better-balanced performance, where 1 signifies perfect prediction, 0 represents random prediction, and −1 indicates complete disagreement between the model’s predictions and actual outcomes. Kappa measures the agreement between a model’s predictions and actual values. A Kappa value of 1 denotes perfect agreement, 0 suggests agreement equivalent to chance, and −1 indicates perfect disagreement.

**Table 1.** Calculated parameters of performance metrics for evaluation of the machine learning model.

Performance metrics	Formula
Accuracy	(TP + TN) / (TP + FN + FP + TN)
Recall	TP / (TP + FN)
Precision	TP / (TP + FP)
F1 score	2TP / (2TP + FP + FN)
MCC	$\frac{\left(\text{TP}\times \text{TN}‐\text{FP}\times \text{FN}\right)}{\sqrt{\left(\text{TP}+\text{FP})(\text{TP}+\text{FN})(\text{TN}+\text{FP})(\text{TN}+\text{FN}\right)}}$

Abbreviations: FN, false negative; FP, false positive; MCC, Matthews correlation coefficient; TN, true negative; TP, true positive.

The paired *t*-test was conducted to compare the performance of the machine learning model between the accuracy during the before cooling phase and the mean accuracy during the after-cooling phase.

## Results

### Trial participants

**Figure 2.**
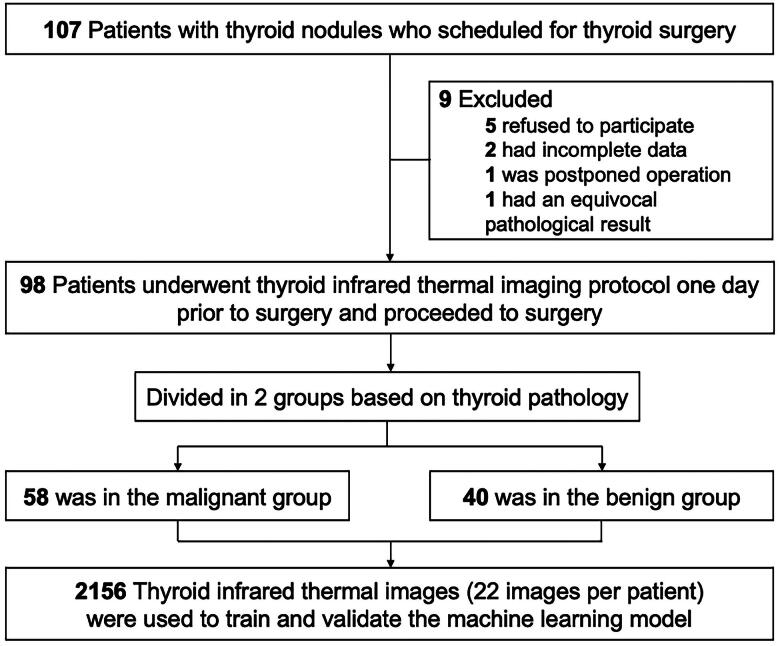
Enrollment and follow-up of the study participants.

A total of 98 participants with a mean age 55.3±1.4 years and 78.6% female, were finally enrolled in the study (Figure 2), after nine participants were excluded due to various reasons: 5 refused to participate, 2 had incomplete data, 1 underwent postponed operation, and 1 had an equivocal pathological result. The participants were divided into two groups based on thyroid pathological results: 58 participants had malignant thyroid nodules (56 papillary thyroid carcinoma and 2 oncocytic thyroid carcinoma) and 40 had benign thyroid nodules. Baseline characteristics are presented in Table 2. There were no significant differences between the malignant and benign groups regarding age, sex, body weight, body mass index (BMI), body temperature, or thyroid function test, including free triiodothyronine (T3), free thyroxine (T4), and TSH. In the malignant group, there were more suspicious ultrasound features according to the American College of Radiology-Thyroid Imaging Reporting and Data System (ACR-TIRADS) and the American Thyroid Association (ATA) classification compared to the benign group (*p* = 0.005 and *p* = 0.001, respectively). Additionally, cytology stratified by the Bethesda system showed a higher percentage of suspicious for malignancy/malignancy in the malignant group than in the benign group (*p* < 0.001). The median nodule size in the malignant group was smaller than in the benign group (1.7 (IQR 0.8–3.2) vs. 3.0 (IQR, 2.4–4.5) cm, *p* = 0.0001).

**Table 2.** Baseline characteristics.

Baseline characteristics	Malignant thyroid nodules (*n* = 58)	Benign thyroid nodules (*n* = 40)	*p*-value
Age, mean (SD), years	54.3 (13.8)	56.6 (13.1)	0.404
Female, *n* (%)	43 (74.1)	34 (85)	0.198
BW, mean (SD), kg	64.5 (13.8)	62.9 (9.3)	0.484
Height, mean (SD), cm	159.3 (7.0)	156.3 (8.2)	0.053
BMI, mean (SD), kg/m^2^	25.3 (4.7)	25.9 (4.6)	0.531
Body temperature, mean (SD), °C	36.5 (0.4)	36.5 (0.4)	0.555
TSH, median (IQR), uIU/mL	1.11 (0.76–1.75)	0.94 (0.49–1.37)	0.067
FreeT4, median (IQR), mcg/dL	0.98 (0.91–1.06)	1.01 (0.94–1.09)	0.252
FreeT3, median (IQR), pg/mL	2.52 (2.39–2.72)	2.63 (2.31–2.80)	0.303
Thyroid imaging, *n* (%)			0.312
Ultrasound	48 (82.8)	30 (75)	
CT	6 (10.3)	9 (22.5)	
MRI	1 (1.7)	0 (0)	
Not performed	3 (5.2)	1 (2.5)	
Ultrasound classification	*n* = 48	*n* = 30	0.005
ACR TIRADS, *n* (%)			
1	0 (0)	1 (3.3)	
2	1 (2.1)	3 (10)	
3	6 (12.5)	11 (36.7)	
4	26 (54.2)	14 (46.7)	
5	15 (31.2)	1 (3.3)	
ATA, *n* (%)			0.001
Very low risk	0 (0)	1 (3.3)	
Low risk	6 (12.5)	13 (43.4)	
Intermediate risk	16 (33.3)	12 (40)	
High risk	26 (54.2)	4 (13.3)	
Cytology (Bethesda system)	*n* = 57	*n* = 36	<0.001
I	4 (7.0)	6 (16.7)	
II	8 (14.0)	24 (66.7)	
III	13 (22.8)	5 (13.9)	
IV	4 (7.0)	0 (0)	
V	8 (14.0)	1 (2.7)	
VI	20 (35.2)	0 (0)	
Nodular size, median (IQR), cm	1.7 (0.8–3.2)	3.0 (2.4–4.5)	0.0001

Abbreviations: ACR, American College of Radiology; ATA, American Thyroid Association; BMI, body mass index; BW, body weight; CT, computed tomography; IQR, interquartile range; MRI, magnetic resonance imaging; SD, standard deviation; T3, triiodothyronine; T4, thyroxine; TI-RADS, thyroid imaging reporting and data system; TSH, thyroid-stimulating hormone.

### Machine learning model performance

**Figure 3.**
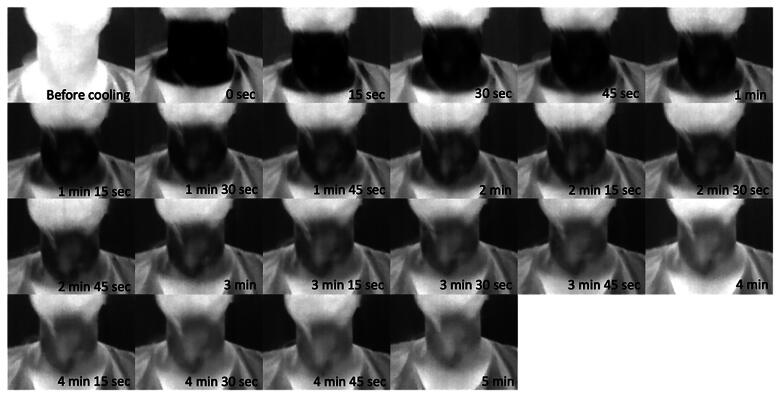
Serial thyroid infrared images over time – example from one participant.

No complications occurred during thyroid infrared thermography. The performance of the machine learning model was evaluated at various time intervals during the thyroid infrared thermography sequence. Table 3 summarizes key performance metrics, including accuracy, recall, precision, F1 score, MCC, Kappa, and the area under the ROC curve. An example of serial infrared thermal images of the thyroid gland was presented in Figure 3. To mitigate the potential confounding effects of nodule size, microcarcinomas (thyroid carcinomas with a diameter less than 1 cm) were excluded, and a repeat analysis was conducted, which continued to demonstrate similar performance metrics.

**Table 3.** Performance metrics of the machine learning model at different time intervals for the initial 10 images.

Parameters		Accuracy	Recall	Precision	F1 score	MCC	Kappa	ROC
Before cooling	Mean	0.75	0.78	0.78	0.76	0.49	0.46	0.63
	SD	0.07	0.26	0.03	0.15	0.15	0.14	0.06
0 s	Mean	0.71	0.73	0.78	0.73	0.39	0.37	0.66
	SD	0.06	0.18	0.12	0.11	0.12	0.12	0.12
15 s	Mean	0.74	0.73	0.81	0.76	0.44	0.42	0.71
	SD	0.07	0.13	0.12	0.08	0.21	0.20	0.13
30 s	Mean	0.61	0.58	0.83	0.60	0.39	0.31	0.57
	SD	0.13	0.32	0.19	0.17	0.10	0.13	0.18
45 s	Mean	0.67	0.51	0.86	0.61	0.39	0.34	0.68
	SD	0.09	0.23	0.15	0.20	0.18	0.17	0.14
1 min	Mean	0.71	0.96	0.68	0.79	0.42	0.33	0.58
	SD	0.10	0.05	0.14	0.09	0.11	0.17	0.18
1 min 15 s	Mean	0.72	1.00	0.69	0.81	0.42	0.32	0.63
	SD	0.13	0.00	0.13	0.09	0.19	0.23	0.18
1 min 30 s	Mean	0.73	0.83	0.75	0.77	0.44	0.41	0.60
	SD	0.08	0.13	0.13	0.07	0.19	0.19	0.10
1 min 45 s	Mean	0.65	0.70	0.80	0.69	0.44	0.36	0.67
	SD	0.12	0.28	0.20	0.12	0.12	0.18	0.17
2 min	Mean	0.67	0.64	0.86	0.68	0.43	0.35	0.64
	SD	0.14	0.29	0.16	0.15	0.23	0.26	0.17
2 min 15 sec	Mean	0.67	0.74	0.80	0.70	0.42	0.32	0.66
	SD	0.13	0.32	0.22	0.15	0.14	0.18	0.19

Abbreviations: Kappa, Kappa hat coefficient; MCC, Matthews correlation coefficient; ROC, Receiver Operating Characteristic.

Before cooling the skin, the model exhibited an accuracy of 0.75 (0.07), indicating moderate accuracy in its predictions. Recall (or sensitivity) was 0.78 (0.26), and with a precision of 0.78 (0.03), demonstrating a reasonable ability to avoid false positives. An F1 score of 0.76 (0.15) suggests a good balance between precision and recall. The MCC of 0.49 (0.15) indicates a moderate correlation between predicted and actual classes, while a Kappa of 0.46 (0.14) suggests moderate agreement. An area under the ROC curve of 0.63 (0.06) suggests a moderate level of discrimination.

There were no significant differences in the performance metrics of thyroid infrared thermographic images during the interval after the cooling phase for detecting malignant thyroid nodules. The accuracy of the machine learning model during the before cooling phase showed a tendency to differ from the mean accuracy during the after-cooling phase, but the difference was not statistically significant (0.75 vs. 0.69, *p* = 0.236).

Our AI model evaluated the transition from using infrared thermographic imaging to a combination of ultrasonographic findings (ACR-TIRADS and ATA classification) and cytology results (Bethesda system) for predicting thyroid malignancy. We observed an increase in accuracy to 0.81 (0.01) and sensitivity to 0.84 (0.1) compared to the results from thyroid infrared thermographic images, which demonstrated an accuracy of 0.75 (0.07) and sensitivity of 0.78 (0.26). The model’s recall of 0.84 (0.10) and precision of 0.85 (0.16) indicate accurate positive predictions. The F1 score of 0.83 (0.05) reflects a balance between precision and recall. The MCC of 0.60 (0.09) shows effective handling of imbalanced datasets, and the Kappa coefficient of 0.55 (0.12) suggests substantial agreement beyond chance. The area under the ROC curve is 0.79 (0.03), indicating good discrimination between classes.

## Discussion

This prospective cross-sectional study provides valuable insights into the potential of convolutional neural network technology for thyroid cancer detection through innovative image processing techniques. A total of 2156 thyroid infrared thermal images from 98 participants were trained and validated by convolutional neural networks. Performance evaluation metrics included accuracy, recall, precision, F1 score, ROC AUC, MCC and Kappa. The machine learning model exhibited good performance in distinguishing benign and malignant thyroid nodules, achieving 75% accuracy and 78% sensitivity before cooling time. However, there was no significant difference in efficacy between the before cooling phase and the after-cooling interval in differentiating thyroid nodules. Furthermore, compared to thyroid ultrasound and cytological results, the convolutional neural network model demonstrated relatively accurate performance in distinguishing thyroid nodules.

An AI application, integrated with infrared thermal imaging, aims to improve thyroid cancer diagnosis. However, when compared to a previous study that did not utilize an AI algorithm and achieved an accuracy of 98.8% and sensitivity of 96.3% using a cutoff point of 2.38 °C [16], our machine learning model, with an accuracy of 75% and a sensitivity of 78% (without using a cutpoint), demonstrates lower performance in thyroid cancer detection. The lack of a clear cutoff point in our study likely led to this reduced performance. Moreover, the previous study using an AI algorithm, achieved an accuracy of 91% [15]. Several factors may explain this discrepancy: differences in the brand and quality of infrared cameras, uncollected confounding factors such as vascular Doppler ultrasonography or insulating factors influences on thermal readings, and potentially, an insufficient sample size for accurate temperature change detection. Additionally, while previous studies used various gold standards such as ultrasound, FNAB, and pathological reports, the present study uses pathological diagnosis as the gold standard.

Thyroid cancer detection through infrared thermography typically utilizes a dynamic acquisition protocol, which integrates external cooling or cold stress methods to enhance the thermal contrasts between a relevant thyroid lesion and the surrounding healthy tissue during the post-cooling thermal recovery phase [13, 15, 16, 21]. Dynamic methods provide an advantage of mitigating the impact of environmental factors, as they do not necessitate the subject to attain a precise steady state, a process that can be very time consuming and challenging. The previous study highlighted the importance of the cooling method in monitoring tumor thermal recovery [13]. However, our study failed to unveil a significant difference in temperature before and after the cooling phase, nor during each time interval of the post-cooling phase. The advancement of machine learning shows potential in refining the dynamic protocol with cooling methods, potentially eliminating redundant steps. Future research should include a comparative study to evaluate the effectiveness of cooling versus non-cooling methods.

Infrared thermal imaging may be also influenced by factors, including the insulating effect of neck fat thickness, variations in nodular size, and the presence of adjacent structures such as the trachea, carotid artery, and jugular vein [22]. Although the present study did not evaluate this insulating effect, one approach to decrease it is by using a 2D or 3D anatomical model derived from computed tomography imaging [22, 23]. Additionally, modern imaging techniques may help eliminate heat interference from adjacent structures such as the carotid artery, by incorporating algorithms designed to compensate for background noise [24].

The strengths of our study include the definite diagnosis of thyroid cancer based on pathological results, as all participants underwent surgery. However, a major limitation is the small sample size, highlighting the need for future studies with larger cohorts to validate the model’s efficacy across more diverse populations. Additionally, the omission of variables that may influence interpretation, such as the thickness of fat layers, distance from the carotid artery, and nodular size, should be addressed in future research. Furthermore, we acknowledge the inherent selection bias, as our study exclusively included patients scheduled for surgery, which may limit the generalizability of our findings. Additionally, this model may not be applicable to certain cases of thyroid nodules, such as individuals with thyroid dysfunction or toxic nodular goiter. Follicular thyroid or medullary thyroid cancers were not observed in this study.

Infrared technology offers user-friendly and safe medical applications, albeit with relatively expensive equipment. Current study protocols are complex and time-consuming. Integrating thermal images into machine learning models may require larger datasets. This technology shows promise as a supplementary tool to enhance the accuracy of detecting thyroid cancer compared to standard methods such as ultrasound and FNAB, as demonstrated by a recent study combining ultrasound and infrared thermal imaging [25]. Further research is needed, particularly regarding its potential integration with ultrasound and FNAB results.

In conclusion, our AI model, trained and validated using noninvasive thyroid infrared thermal images, demonstrates good accuracy in diagnosis of thyroid cancer. It serves as a complementary tool for the assessment of thyroid nodules, particularly in cases where FNAB results are inconclusive or unavailable.

## Data Availability

The data sets generated and analyzed during this study are available from the corresponding author upon request.
